# Identification of key biomarkers and immune infiltration in the thoracic acute aortic dissection by bioinformatics analysis

**DOI:** 10.1186/s12872-023-03110-4

**Published:** 2023-02-08

**Authors:** Jun Luo, Haoming Shi, Haoyu Ran, Cheng Zhang, Qingchen Wu, Yue Shao

**Affiliations:** 1 Department of Cardiothoracic Surgery, The First Affiliated Hospital of Chongqing Medical University, No. 1 Youyi Road, Yuzhong District, Chongqing, China; 2 Chongqing Medical University, Chongqing, China

**Keywords:** Bioinformatics analysis, Thoracic acute aortic dissection, Differentially expressed genes, Immune-inflammatory responses

## Abstract

**Background:**

Thoracic acute aortic dissection (TAAD), one of the most fatal cardiovascular diseases, leads to sudden death, however, its mechanism remains unclear.

**Methods:**

Three Gene Expression Omnibus datasets were employed to detect differentially expressed genes (DEGs). A similar function and co-expression network was identified by weighted gene co-expression network analysis. The least absolute shrinkage and selection operator, random forest, and support vector machines-recursive feature elimination were utilized to filter diagnostic TAAD markers, and then screened markers were validated by quantitative real-time PCR and another independent dataset. CIBERSORT was deployed to analyze and evaluate immune cell infiltration in TAAD tissues.

**Results:**

Twenty-five DEGs were identified and narrowed down to three after screening. Finally, two genes, SLC11A1 and FGL2, were verified by another dataset and qRT-PCR. Function analysis revealed that SLC11A1 and FGL2 play significant roles in immune-inflammatory responses.

**Conclusion:**

SLC11A1 and FGL2 are differently expressed in aortic dissection and may be involved in immune-inflammatory responses.

**Supplementary Information:**

The online version contains supplementary material available at 10.1186/s12872-023-03110-4.

## Introduction

Thoracic acute aortic dissection (TAAD) is a life-threatening disease with high mortality of 2.6–3.6 cases per 100,000 per annum [1]. The risk of death for patients with TAAD increases by 1% per hour before medical and surgical intervention [2], with 40–50% mortality in 48 h. However, the potential molecular mechanisms of TAAD remain unclear, and there is currently no effective medicine to control or alleviate TAAD progression or development.

As bioinformatics develop, increased cardiovascular diseases, including acute myocardial infarction [3] and heart failure [4], are better understood. However, there are few related studies to aortic dissection (AD), so this study aimed to identify significant TAAD DEGs through bioinformatics analysis to provide directions for diagnosis and therapy.

## Methods

### Gene expression datasets

Three datasets (GSE52093, GSE98770, and GSE147026), including gene expression profiles of TAAD (23 patients) and controls (19 healthy people), were downloaded from the Gene Expression Omnibus database (GEO, http://www.ncbi.nlm.nih.gov/geo). The normal aortic tissue samples were obtained from patients undergoing coronary artery bypass grafting surgery (CABG) or obtained from donors without any aortic diseases. The aortic tissue of AD was obtained from patients undergoing an ascending aortic replacement surgery during a cardiopulmonary bypass. In addition, we use GSE153434 (10 TAD patients and 10 healthy controls) for external validation. Additional file 1: Table S1 summarized the detailed information of all the datasets used in this study.

### Clinical samples

Twenty TAAD samples were obtained from patients admitted for surgery to the First Affiliated Hospital of Chongqing Medical University from June 2021 to March 2022, and 15 healthy control aorta samples were obtained from organ donors without any vascular diseases. The details of the studied population characteristics are presented in Table 1.

**Table 1:** Baseline data of all patients

Characteristic	NA	AD	*p*
n	15	20	
Gender, n (%)			0.565
Female	2 (13.3%)	1 (5%)	
Male	13 (86.7%)	19 (95%)	
Obesity (BMI > 25 kg/m^2^), n (%)			1.000
No	5 (33.3%)	7 (35%)	
Yes	10 (66.7%)	13 (65%)	
Diabetes, n (%)			1.000
No	14 (93.3%)	19 (95%)	
Yes	1 (6.7%)	1 (5%)	
Hypertension, n (%)			0.037
No	12 (80%)	8 (40%)	
Yes	3 (20%)	12 (60%)	
Smoking, n (%)			0.721
No	6 (40%)	6 (30%)	
Yes	9 (60%)	14 (70%)	
Drinking, n (%)			1.000
No	12 (80%)	15 (75%)	
Yes	3 (20%)	5 (25%)	
Family history of aortic diseases, n (%)			0.496
No	15 (100%)	18 (90%)	
Yes	0 (0%)	2 (10%)	
Age, mean ± SD	41.87 ± 9.75	44.05 ± 5.84	0.415

*NA* Normal artery, *AD* Aortic dissection, *BMI* Body mass index, *SD* Standard deviation

### Identification of differentially expressed genes (DEGs)

Three TAAD and healthy controls datasets were merged, and the batch effect was removed by ComBat in the “sva” R package. The “Limma” package was used to normalize the mRNA expression data, and a principal component analysis (PCA) plot was constructed through the “ggplot2” package. DEGs were identified with thresholds of |log2fold-Change(FC)|> 1.0 and adjusted *p* value < 0.05. The heatmap represents the relative DEGs expression levels.

### Weighted gene co-expression network analysis (WGCNA)

WGCNA allows biologically meaningful module information mining based on pairwise correlations between genes in high-throughput data. The co-expression network was constructed by the 25% top genes with the highest expression variance. The adjacency matrices storing the whole co-expression network information were calculated using Pearson’s correlation matrices. The average linkage hierarchical clustering method was performed for clustering dendrograms with a minimum module size of 30 based on the topological overlap measure (TOM) matrices. Finally, similar gene modules were merged with a threshold of 0.25, and the most significantly different modules in the AD compared to normal tissues were used to screen key genes.

### Screening of diagnostic markers

The support vector machine recursive feature elimination (SVM-RFE), least absolute shrinkage selection operator (LASSO), and random forest were performed to identify more significant DEGs. LASSO was applied with the “glmnet” package. SVM-RFE is a mode to identify the most variables by deleting SVM-generated eigenvectors based on the “e1071” package. The random forest was performed by the “randomForest” package. Finally, the results were merged to obtain the core genes.

### Identification of genes by clinical samples

DEGs expression was evaluated using RT-qPCR. Total RNA was reversed to cDNA using PrimeScript RT reagent Kit (TaKaRa, Japan) according to the manufacturer’s instructions. All relevant primers are listed in Additional file 1: Table S2. All samples were normalized to GAPDH.

### Gene ontology (GO), gene set enrichment analysis (GSEA), and analysis of immune cell infiltration

GO enrichment analyses were performed with the cluster Profiler package on the screened genes from DEGs and WGCNA combination (selected with enrichment significance evaluated at *p* < 0.05). GSEA software (Broad Institute, Cambridge, MA, USA) was used to explore the functional characteristics of individual genes, FGL2 and SLC11A1, via the “cluster profile” package of R software [5–7]. Moreover, CIBERSORT was used to analyze the infiltration of immune cells of AD and selected DEGs. All immune cells were plotted using the “vioplot” R package, and each gene-related immune cell was plotted using the “corrplot” R package.

### Statistical analysis

All statistical analyses were performed by R software (R version 4.1.0; https://www.r-project.org/). Baseline patient data were analyzed using Fisher’s exact test and Student’s t-test. A *p* value < 0.05 was considered significant statistically.

## Results

### DEGs screening

**Fig. 1:**
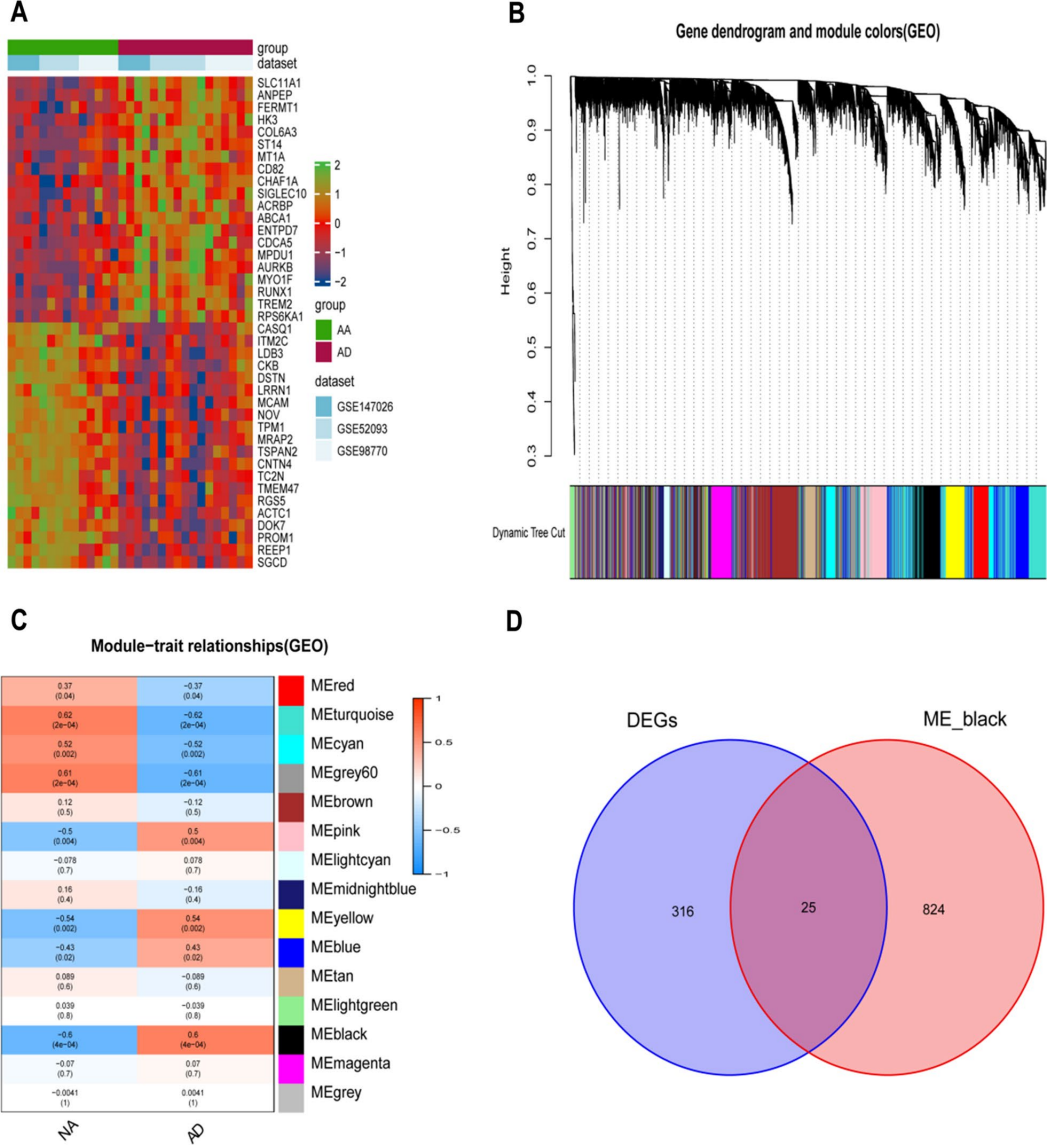
Differentially expressed genes and WGCNA. **A** Heat maps shows the top 20 up-regulated and top 20 down-regulated genes between NA and AD tissue. **B** Dendrogram of the gene modules. Te branches represent different gene modules, and each leaf represents a gene in the cluster dendrogram. **C** Correlation heat map of gene modules and AD, the black is positively correlated with AD. **D** The Venn graph shows intersection of DEGs and genes in black module. *WGCNA* Weighted gene co-expression network analysis, *NA* Normal artery, *AD* Aortic dissection, *DEGs* Differentially expressed genes

In total, 340 DEGs were identified from the three GEO datasets of TAAD, of which 156 genes were upregulated, and 184 were downregulated. The clustered heat map (Fig. 1A) of all top 20 up-regulated and top 20 down-regulated genes was performed to distinct differences between normal and AD tissues.

### WGCNA and identification of key modules

A dendrogram of TAAD samples was clustered using Pearson's correlation method and the average linkage method. Co-expression analysis was performed to construct the expression network. The dendrogram of all differentially expressed genes was performed based on whether there was an AD, and the co-expression network was analyzed based on the optimal soft threshold, with the genes divided into different modules by a gene cluster tree (Fig. 1B). 15 modules were identified using hierarchical clustering in Fig. 1C, with the black module containing 849 genes being the most significantly different between AD and normal tissues with a favorable correlation. The merging of DEGs and WGCNA obtained 25 genes (Fig. 1D).

### GO enrichment analyses

JAK2, SLC11A1, and FGL2 genes were associated with four special functional GO terms, GO0002604, GO0002468, GO0045342, and GO0002577 (Table 2). Most of these were related to antigen processing and presentation, implying that the three genes have important roles in immunoreaction. Combing the three genes revealed that two genes, SLC11A1 and FGL2, also overlapped in their functional aspects.

**Table 2:** GO analyses results of DEGs

ID	Description	GeneRatio	BgRatio	*p* value	p.adjust	q value
GO:0002604	Regulation of dendritic cell antigen processing and presentation	2/22	11/18670	7.24e−05	0.033	0.025
GO:0002468	Dendritic cell antigen processing and presentation	2/22	12/18670	8.69e−05	0.033	0.025
GO:0045342	MHC class II biosynthetic process	2/22	15/18670	1.38e−04	0.035	0.027
GO:0002577	Regulation of antigen processing and presentation	2/22	20/18670	2.49e−04	0.047	0.036

*GO* Gene ontology, *DEGs* Differentially expressed genes

### Identification of key biomarkers

**Fig. 2:**
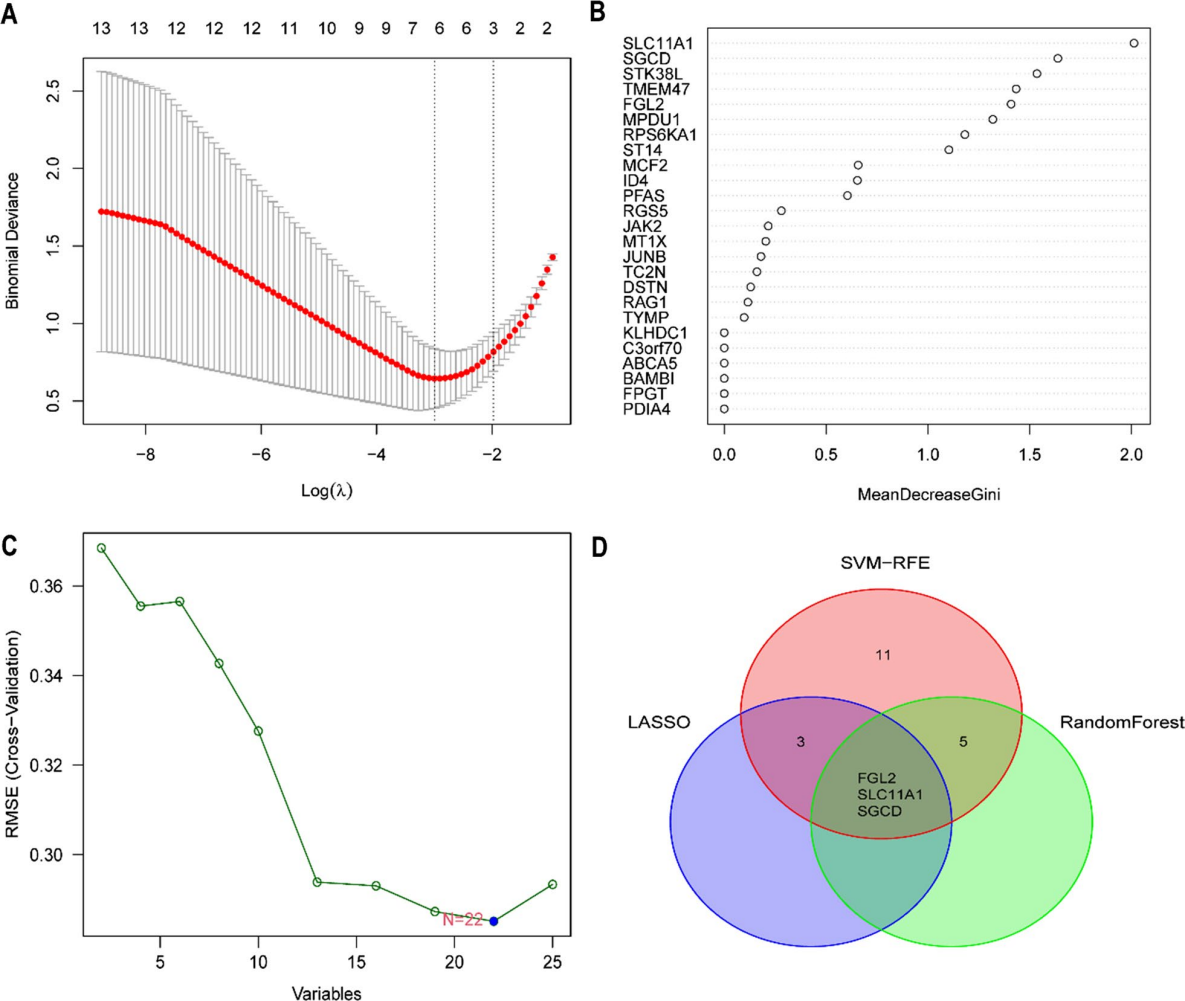
Screening diagnostic markers based on LASSO, random forest and SVM-RFE. **A** The LASSO logistic regression algorithm to screen diagnostic markers. **B** The top 25 significant genes recognized from random forest analysis. **C** Feature selection based on the random forest algorithm. The x-axis indicates the number of used variables. The y-axis is the cross-validation error of each prediction model. **D** The intersected genes of these three analyses were selected. *LASSO* Least absolute shrinkage and selection operator, *SVM-RFE* Support vector machines-recursive feature elimination

The LASSO, random forest, and SVM-RFE were used to filter diagnostic TAAD markers (Fig. 2A–C). Three screening identified genes named FGL2, SLC11A1, and SGCD (Fig. 2D).

### Verification using GEO dataset and tissue samples

**Fig. 3:**
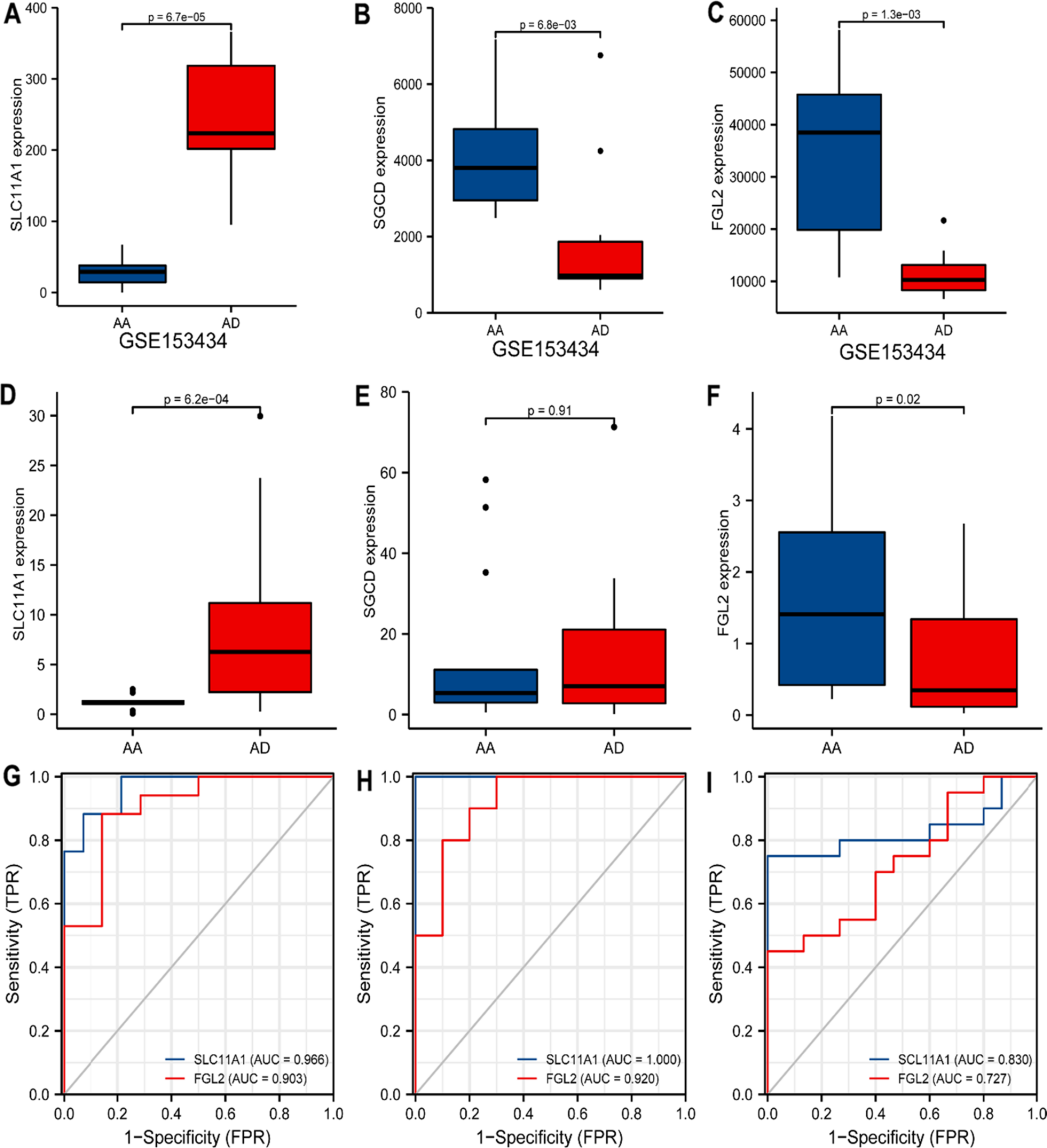
Verification of gene expression analysis and ROC analyses. The expression of the SLC11A1 (**A**), SGCD (**B**), and FGL (**C**) in validation set (GSE15343). RT-PCR validation of the SLC11A1 (**D**), SGCD (**E**), and FGL2 (**F**) between AD and normal controls. **G** The ROC curve of the diagnostic efficacy verification training set. **H** The ROC curve of the diagnostic efficacy verification in validation set (GSE15343). **I** The ROC curve of the diagnostic efficacy verification in qPCR samples. *AD* Aortic dissection, *ROC* Receiver operating characteristic curve

The GEO dataset GSE153434, containing ten normal samples and diseased aorta of TAAD, was used to verify the intersected genes, showing that the three genes FGL2, SLC11A1, and SGCD have the same tendency in diseased and normal samples (Fig. 3A–C). The differential expression was verified in clinical samples showing that only FGL2 and SLC11A1 have the same trend as the dataset analysis; FGL2 was highly expressed in TAAD, whereas SLC11A1 was less expressed (Fig. 3D–F). The diagnostic sensitivity of these two genes was evaluated with ROC curves from the merged dataset, validated dataset, and tissue samples, demonstrating that SLC11A1 and FGL2 had scores greater than 0.7, implying perfect diagnostic sensitivity but SLC11A1 had better sensitivity (Fig. 3G–I).

### Immune cell infiltration and gene set enrichment analysis (GSEA)

**Fig. 4:**
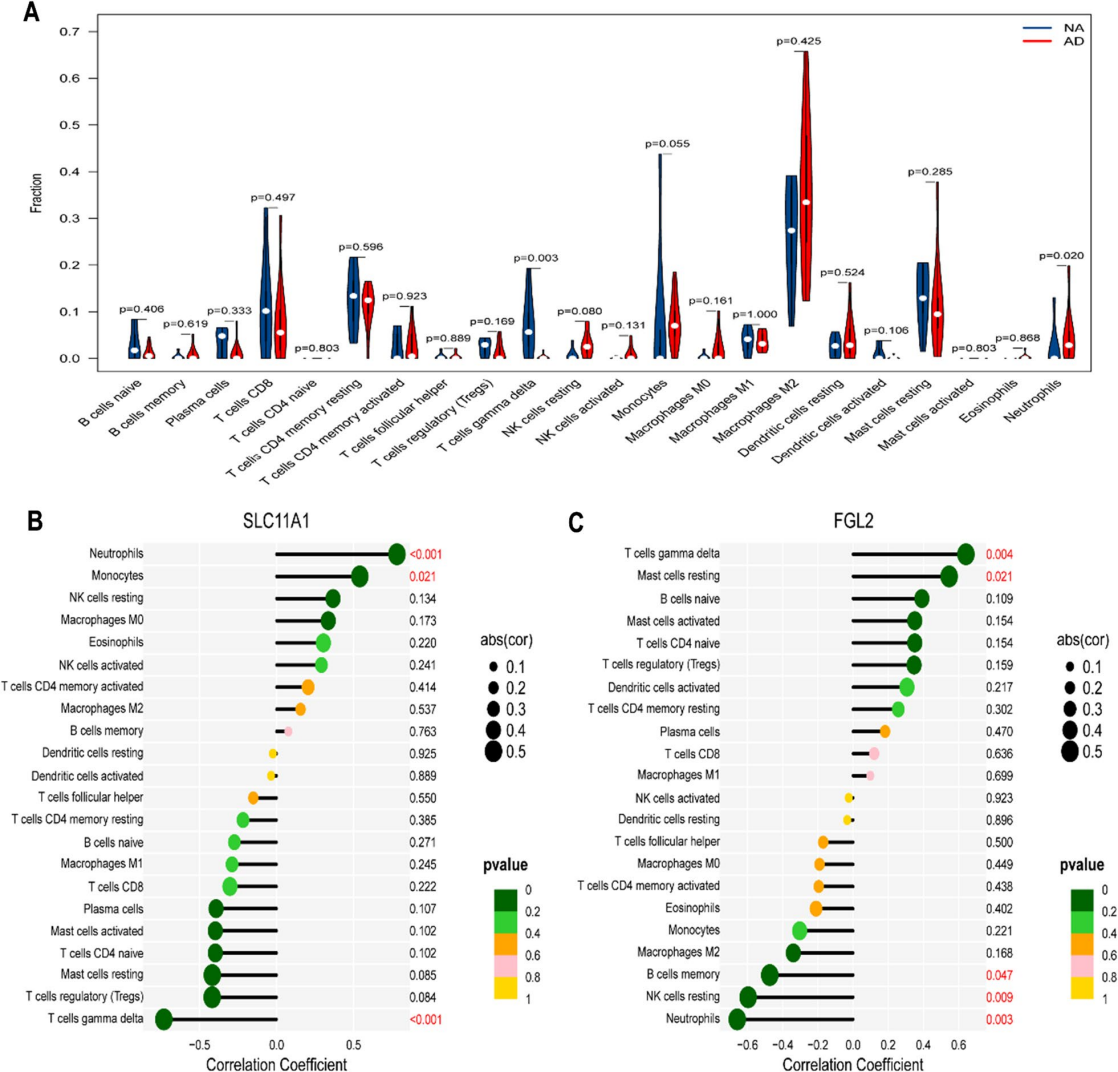
The landscape of immune infiltration and correlation between SLC11A1, FGL2, and infiltrating immune cells. **A** The difference of immune infiltration between AD tissue and normal controls. **B** Correlation between SLC11A1 and infiltrating immune cells. **C** Correlation between FGL2 and infiltrating immune cells. AD, aortic dissection

**Fig. 5:**
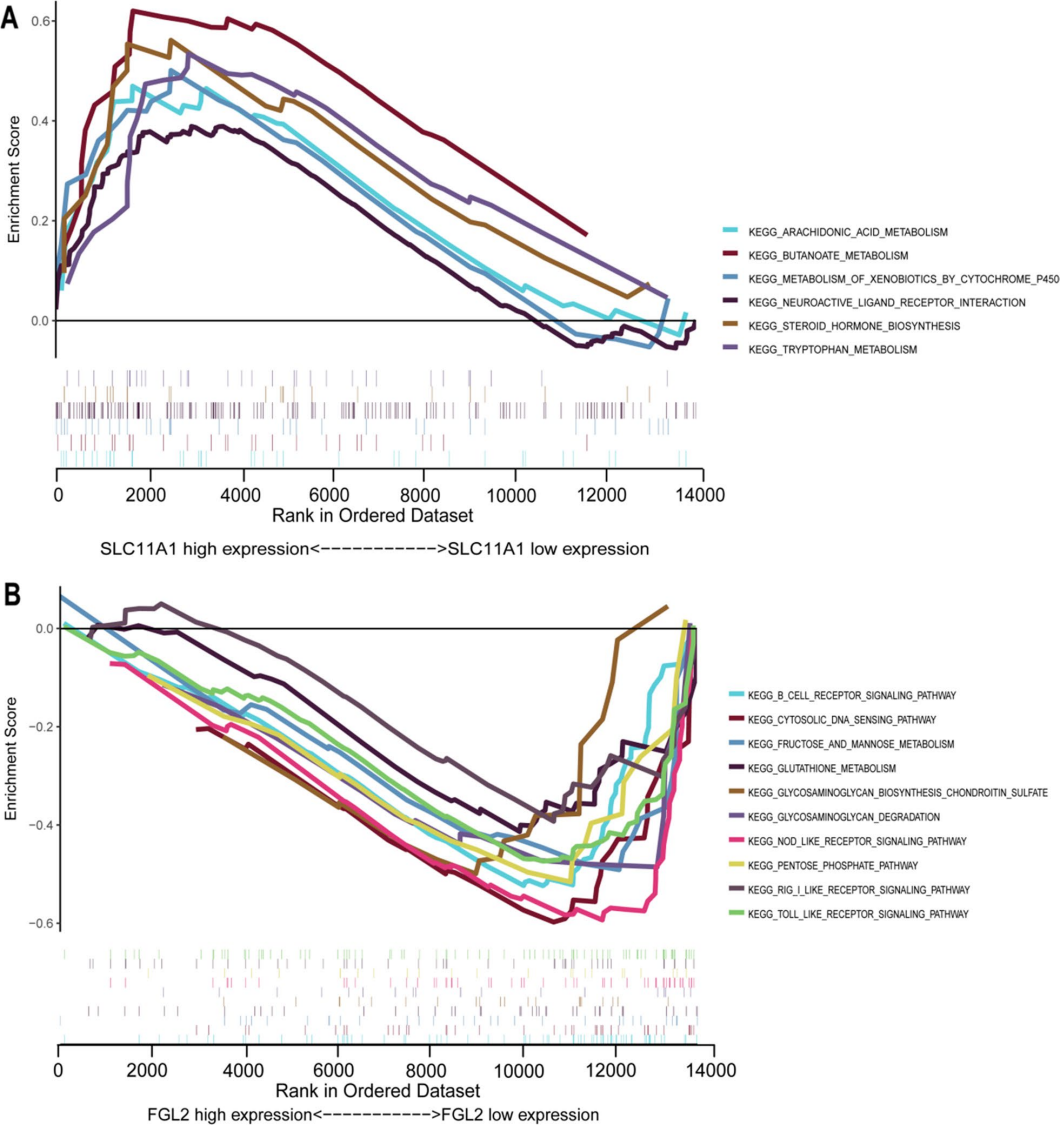
Result of gene set enrichment analysis. **A** KEGG pathways (http://www.genome.jp/kegg/) enriched in SLC11A1. **B** KEGG pathways enriched in FGL2. *KEGG* Kyoto encyclopedia of genes and genomes

The immune cells, resting NK cells, monocytes, and neutrophils were more highly expressed in AD, whereas Tregs and γδT cells had lower expression (Fig. 4A). The analysis of each immune cell and individual genes revealed that SLC1A11 was linked to a higher level of neutrophils and monocytes but a lower level of regulatory T cells and γδT cells. However, FGL2 had a similar trend to γδT cells and resting mast cells but the opposite trend to neutrophils and resting NK cells (Fig. 4B,C). Also, GSEA enrichment analysis revealed that SLC11A1 was associated with metabolic pathways, whereas FGL2 was relevant to B humoral cellular immunity and inflammation (Fig. 5A,B).

## Discussion

Three AD datasets were screened to identify 25 DEGs, of which FGL2 and SLC11A1 were verified to be associated with immuno-inflammatory responses in TAAD.

AD is associated with a high immune-inflammation level which destroys and weakens the normal function of the aortic wall and leads to rupture. Immune cells, including neutrophils, NK, and B cells, are significantly increased in the peripheral blood of AAD patients [8]. Moreover, macrophages and activated B and T lymphocytes were proved to accumulate inside the aortic wall around the vasa vasorum and at the edge of the ruptured media [9, 10]. In addition, upregulation of pro-inflammatory cytokines and has been described in patients with type-A Stanford AD [11].

The neutrophil results were consistent with other studies [12–14]. Tissue damage in AD can induce local inflammation and secrete damage-related molecular patterns (DAMP), including proteases and cytokines, thereby promoting early neutrophil recruitment and activation [15]. Neutrophil hyperactivity can lead to tissue damage in severe inflammation, explaining the high expression of neutrophils in AD.

γδT cells, a subset of the T cell family, are shown to be distributed on abundant lymphatic, blood, epithelial and mucosal surfaces [16]. The function of γδT cells is complex, and the role of γδT cells in the development of aortic dissection remains unclear. Many studies exhibited γδT cells with immune function secreting various cytokines (IL-17 and IFN-γ), influencing immune cell recruitment [17–19].

Interestingly, our study found low abundance of γδT cells in aortic dissection vessel tissue. This is in accordance with several other studies which also found low levels of γδT cells, although some were not statistically significant [20–23]. We think the cause may be as follows. γδT cells tend to bind to and be activated by aortic endothelial cells [24, 25]. Massive endothelial cell apoptosis and intima denudation are essential for the onset of dissection [26], which may lead to a reduction in γδT cells can be detected.

Fibrinogen-like 2 (FGL2) is a member of the fibrinogen-associated protein superfamily. Previous studies found that FGL2 has immunosuppressive effects on adaptive immunity by inhibiting dendritic cell maturation, downregulating T cell function and inducing B cell apoptosis [27], and the present study found that it is associated with infiltration of neutrophils and NK cells in AD. FGL2 is mainly derived from regulatory T cells (Tregs), and it reduces the production of IFN-γ and IL-17 [13, 28]. Whereas regulatory T cells have low expression in AD [19, 29], IFN-γ and IL-17 are highly expressed in AD [19, 30]. This suggests that the low expression of FGL2 in AD may be caused by a decrease in regulatory T cells and mediated by IFN-γ and IL-17 to promote AD.

The solute carrier family 11 member a1 protein (SLC11A1) exerts multiple effects on immune-inflammatory functions, including enhanced antigen presentation to T cells, overexpression of MHC class II, increased production of pro-inflammatory cytokines and upregulation of neutrophil chemokines [31–33]. In addition, SLC11A1 is expressed in macrophages and neutrophils and can regulate macrophage and neutrophil infiltration in arthritis and colitis [34, 35]. In our study, we found that SLC11A1 was highly expressed in AD and positively correlated with neutrophil and macrophage infiltration. The high expression of SLC11A1 may aggravate the inflammatory damage in AD by promoting neutrophil and macrophage infiltration.

The present study has several limitations. First, the datasets and clinical tissues were insufficient, and although two specifically expressed genes were identified, further studies are required to verify the findings. Second, the protein expression was undetected and unverified, so the specific mechanisms involved in the immune-inflammatory response in AD require further study. In this study, various bioinformatics methods were utilized to select the key genes with good diagnostic values, which were further verified by clinical samples. These results which contain multiple datasets from multiple centers are credible. Moreover, we found that FGL2 and SLC11A1 are associated with immune infiltration in AD, which sets the stage for future mechanistic exploration.

## Conclusion

Two genes, FGL2 and SLC11A1, were significantly differentially expressed in AD and were involved in immune inflammation.

## Supplementary Information

**Additional file 1. Table S1.** The detail information of GSE datasets. **Table S2.** Primer sets used in the present study.

## Data Availability

GEO data can be obtained from the official website (https://www.ncbi.nlm.nih.gov/geo). The datasets used and/or analyzed during the current study are available from the corresponding author on reasonable request.
