# Supplementary material for: Identification of key biomarkers and immune infiltration in the thoracic acute aortic dissection by bioinformatics analysis
Source: BMC Cardiovasc Disord. 2023 Feb 8;23:75. doi: 10.1186/s12872-023-03110-4 (PMC9909862; doi:10.1186/s12872-023-03110-4)
Supplement: Supplementary file 1 — Additional file 1. Table S1. The detail information of GSE datasets. Table S2. Primer sets used in the present study. [file 12872_2023_3110_MOESM1_ESM.doc]

**Supplementary table 1.** The detail information of GSE datasets.

| Dataset ID | Cases of NA | Cases of AD | Platform | region |
| --- | --- | --- | --- | --- |
| GSE52093 | 5 | 7 | GPL10558 | China |
| GSE98770 | 5 | 6 | GPL14550 | Japan |
| GSE147026 | 4 | 4 | GPL24676 | China |
| GSE153434 | 10 | 10 | GPL20795 | China |

Abbreviations: NA, normal artery; AD, aortic dissection.

**Supplementary table 2. Primer sets used in the present study**

| **Gene names** | **5’-3’** | **Sequences** |
| --- | --- | --- |
| 18s | Forward | AAACGGCTACCACATCCAAG |
| 18s | Reverse | CCTCCAATGGATCCTCGTTA |
| SLC11A1 | Forward | CTTCAGCCTGCGGAAGCTAT |
| SLC11A1 | Reverse | TCTGACTCGATGTTTCCTGGG |
| SGCD | Forward | AGGGCACAGTGTTCCCTAAAT |
| SGCD | Reverse | GGGGCCTCCATCACTAGAGA |
| FGL2 | Forward | AGATTGCTCTGACTACTACGCA |
| FGL2 | Reverse | TGCCATGTTCTGGTGAAGTTG |
